# Scanning X-ray nanodiffraction: from the experimental approach towards spatially resolved scattering simulations

**DOI:** 10.1186/1556-276X-7-553

**Published:** 2012-10-06

**Authors:** Martin Dubslaff, Michael Hanke, Jens Patommel, Robert Hoppe, Christian G Schroer, Sebastian Schöder, Manfred Burghammer

**Affiliations:** 1, Paul-Drude-Institut für Festkörperelektronik, Hausvogteiplatz 5-7, Berlin, D-10117, Germany; 2, Technische Universität Dresden, Institut für Strukturphysik, Zellescher Weg 16, Dresden, D-01069, Germany; 3, European Synchrotron Radiation Facility, Grenoble Cedex, F-38043, BP 220, France

**Keywords:** Quantum dots, Quantum dot molecules, X-ray nanodiffraction, X-ray scattering simulation, 68.65.Hb, 61.05.C-

## Abstract

An enhancement on the method of X-ray diffraction simulations for applications using nanofocused hard X-ray beams is presented. We combine finite element method, kinematical scattering calculations, and a spot profile of the X-ray beam to simulate the diffraction of definite parts of semiconductor nanostructures. The spot profile could be acquired experimentally by X-ray ptychography. Simulation results are discussed and compared with corresponding X-ray nanodiffraction experiments on single SiGe dots and dot molecules.

## Background

At synchrotron facilities, high-resolution X-ray diffraction with nanofocused beams (nHRXRD) is emerging as a new tool to analyze semiconductor nanostructures. Thereby, X-ray beams focused down to 100 nm provide the advantage of illuminating single nanostructures or individual parts of them with hard X-rays at high photon flux. Using nHRXRD, different nano-objects like quantum dots (QDs) or small ensembles of QDs, so-called quantum dot molecules (QDMs), as well as nanowires, step edges, ridges, or more complex electronic devices, can be examined individually by diffraction [1-7].

**Figure 1:**
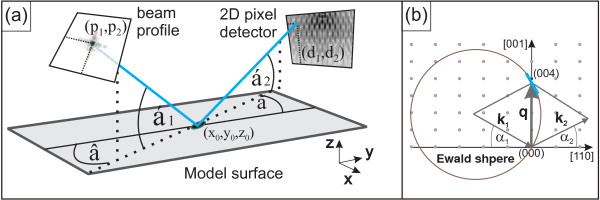
**Sketch of the geometrical situation and variable definitions.***p*_1_,*p*_2_are the beam profile pixel coordinates; *α*_1_and *α*_2_are the beam incidence and exit angles with related azimuth angles *β*and *γ*. *d*_1_,*d*_2_are the detector pixel coordinates, and *r*_0_=(*x*_0_), *y*_0_, *z*_0_) is the reference point of the spot center at the sample surface **(a)**. A 2D sketch of the situation in reciprocal space for the (004) reflection is shown in **(b)**. Therein, **k**_1_, **k**_2_are the wave vectors, **q** is the scattering vector, and the 2D detector (blue line) stands orthogonally to the sketch (which is the [$1\bar{1}0$] direction).

Practically, these experiments with highly focused beams are performed by scanning the sample surface while recording diffraction images with a two-dimensional (2D) detector. The captured images represent reciprocal space maps (RSMs) showing 2D slices through the three-dimensional (3D) reciprocal space (see also Figure 1b). The speckle pattern and diffuse surrounding of Bragg reflections in RSMs can then reveal positional ordering of objects, shape of structures, or strain field within the investigated material [4,8,9]. 

Often, scattering simulations based on elastic strain analysis serve as a very valuable method to support or even find interpretations of the experimentally obtained RSMs [10-12]. However, up to now, the illumination of individual parts of nanostructures, the characteristic feature of nHRXRD, has commonly not been taken into account in the scattering simulations. In this paper, we present nHRXRD measurements and a more sophisticated simulation approach dedicated to nanodiffraction applications, which combines the illumination function of the primary X-ray beam and finite element method (FEM) with a kinematical scattering scenario.

## Methods

### X-ray scattering simulations

Initially, a 3D model of the nanostructure is created, which is based on shape information derived from direct scanning probe techniques. Further, we perform FEM calculations on the model. The FEM considers the full elastic anisotropy and the lattice mismatch governed by the chemical composition. Finally, the displacement field **u** derived by FEM [13] may serve as an input for the kinematical scattering simulations (see further below).

As a second input for our nanofocus scattering simulations, we use a beam spot profile which, on the one hand, can be set to any artificial intensity distribution for the desired spot shape. On the other hand, the spot profile can be obtained experimentally on the basis of the actual illumination function, e. g., by X-ray ptychography. Formerly developed for electron microscopical applications [14,15], ptychography has been rediscovered for the purpose of scanning X-ray microscopy during the last years [16-19]. Ptychography consists of a measurement where a coherent focused beam scans the sample with a step size smaller than the beam diameter, which leads to a spatial overlap of the successively illuminated areas. For each spot position, the scattered intensities are captured by a 2D detector in far-field geometry. From the redundant information about the scanned object within the recorded scattering intensities, due to the spot overlap, the real space image of the object’s structure and the 3D wave field of the focused beam can be calculated.

**Figure 2:**
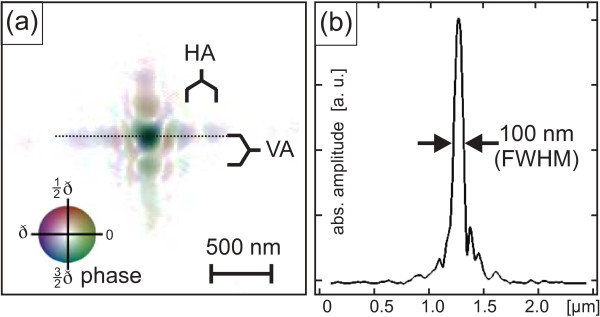
**X-ray beam focused by nanofocusing lenses.(a)** shows the illumination function of an X-ray beam which has been obtained by X-ray ptychography prior to a nanofocus diffraction experiment [6]. The image of the illumination function depicts a cross section of the amplitude and phase within the focal plane of the beam and, thus, determines the final spot profile at the sample surface. HA and VA indicate side oscillations due to the lens apertures. **(b)** is a projection of the intensity distribution of the illumination function to the horizontal axis (dotted line in (a)).

A picture of an illumination function is shown in Figure 2, which was gained using ptychography before performing one of the diffraction experiments. To characterize the nanobeam, a ptychogram of a test sample was taken (for details on such a measurement, see [20]). Figure 2a depicts the focal plane within the beam caustic, whereas Figure 2b shows a horizontal line cut through the center of Figure 2a revealing a beam size of 100 nm full width at half maximum (FWHM). Besides the amplitude values (pixel brightness), Figure 2a also contains information about the phase within the beam encoded by the hue value (see scale). As focusing optics for hard X-rays, we used refractive nanofocusing lenses made of silicon [8]. The cross-like vertical and horizontal oscillations VA and HA in Figure 2a are diffraction phenomena because of the lens aperture and appear slightly axially rotated corresponding to a tilt of the lenses (visible at the dotted line in Figure 2a) [20]. 

In the scattering simulations, we realized the nanofocus spot by introducing a set of weighting factors, 0≤*w*_*n*_≤1, that determine the amplitude of the incident beam for each scatterer *n*, *n*∈*N* with 1≤*n*≤*N*, *N* number of scatterers. The factors *w*_*n*_are calculated from the brightness of each RGB pixel within the beam profile image (e. g., Figure 2) and, thus, act mathematically as a ‘finite support’ which selects only scatterers within the sample model that should be illuminated by the nanofocused beam. With this approach, beam profiles of different origins can be handled, e.g., experimentally gained profiles (Figure 2) or artificially created profiles (for instance, perfect disks or rectangles). The 3D model of the FEM calculations has been used as an input, which yields the positions of each scatterer **r**_*n*_ = (*r*_*x*,*n*_,*r*_*y*,*n*_,*r*_*z*,*n*_) as well as the displacement field **u**(**r**_*n*_) caused by strain relaxation.

To calculate *w*_*n*_for each scatterer within the 3D model, it is necessary to correlate the positions of the scatterers **r**_*n*_ with the pixel coordinates *p*_1_, *p*_2_of the 2D beam profile. Using the geometry and angle definitions illustrated in Figure 1a, this has been done by a projective transformation formula as follows (in round numbers):

(1) $$\begin{array}{ll} p_{1}= & s((r_{y,n}-y_{0})\cos(\beta )-(r_{x,n}-x_{0})\sin(\beta ))+c_{1}\\ p_{2}= & s[\sin(\alpha_{1})((r_{x,n}-x_{0})\cos(\beta )+(r_{y,n}-y_{0})\sin(\beta ))\\ & +(r_{z,n}-z_{0})\cos(\alpha_{1})\;]+c_{2} \end{array}$$

Scaling factor *s* in units of ‘pixels per model length unit’ correlates the length scales of the model and the beam profile; thus, *s* also sets the simulated beam spot size. Constants *c*_1_ and *c*_2_ define the beam center within the beam profile pixel array.

Using Equation 1, the pixel brightness *b*(*p*_1_,*p*_2_) can be identified with *b*(*r*_*n*_). Considering a spot brightness minimum *b*_*min*_ and maximum *b*_*max*_, we obtain a weighting factor *w*_*n*_=*b*(*r*_*n*_)/(*b*_*max*_−*b*_*min*_) for each scatterer *n*. In case Equation 1 yields values for *p*_1_,*p*_2_ that are beyond the beam profile pixel array, *w*_*n*_is set to zero. The beam phase *ϕ*_*n*_at each scatterer is calculated from the beam profile as well. For that, the pixel hue value *h*_*n*_↦*h*(*p*_1_,*p*_2_) is multiplied by the number of periods resulting from the distance between scatterer *n* and a fixed plane orthogonal to the beam path in units of the wave length *λ*:

(2) $$\begin{array}{ll} \phi_{n}=\frac{h_{n}}{\lambda } & \left[\;\cos(\alpha_{1})(r_{x,n}\cos(\beta )+r_{y,n}\sin(\beta )\right)\\ & +r_{z,n}\sin(\alpha_{1})\;] \end{array}$$

If we include the phase *ϕ*_*n*_and weighting factors *w*_*n*_into the coherent sum of the kinematical scattering, e. g. reference [11], the intensity results in:

(3) $$I\propto {\left|\overset{N}{\underset{n=1}{\sum }}\;w_{n}e^{i\phi_{n}}e^{i\text{q}({\text{r}}_{n}+\text{u}({\text{r}}_{n}))}\right|}^{2}$$

The sum runs over all N ‘basic cells’ which the model is consist of (see [11]). Multiple scattering, refraction, and absorption within the crystal have been neglected. The values for the scattering vector **q** are calculated from the incidence angle *α*_1_ and the corresponding azimuth angle *β* as well as the exit angle for diffusely scattered intensities $\left(\alpha_{2}^{\prime }\right)$ with its respective azimuth angle *γ*^′^:

(4) $$\text{q}=\frac{2\Pi }{\lambda }\left(\begin{array}{cc} \cos(\overset{\prime }{\underset{2}{\alpha }})\;\cos(\gamma^{\prime }) & -\cos(\alpha_{1})\;\cos(\beta )\\ \cos(\overset{\prime }{\underset{2}{\alpha }})\;\sin(\gamma^{\prime }) & -\cos(\alpha_{1})\;\sin(\beta )\\ \sin(\overset{\prime }{\underset{2}{\alpha }})\;\; & +\;\;\;\sin(\alpha_{1}) \end{array},\;\right)$$

wherein the angles $\left(\alpha_{2}^{\prime }\right)$ and *γ*^′^of diffuse scattering are derived geometrically from the specular beam (at *α*_2_, *γ*) and the position of the detector pixels (*d*_1_, *d*_2_) in 3D real space. We applied the nanospot diffraction simulation to SiGe/Si(001) islands, SiGe/Si(001) dot molecules, and InGaAs/GaAs(001) quantum dot molecules using spot sizes of 200, 250, and 100 nm, respectively (see the succeeding sections).

## Results and discussion

### Individual SiGe/Si(001) islands

As a first experimental demonstration [2], we used single Si_0.85_Ge_0.15_/Si(001) islands with a base length of 1.2 *μ*m since these SiGe structures emerged as excellent test objects for our nanofocus experiments as well as the nanofocus simulations. The X-ray spot size amounted to 200 nm (FWHM) focused by refractive nanofocusing lenses at a photon energy of 15.25 keV. All nanofocus experiments presented here were performed at beamline ID13, European Synchrotron Radiation Facility (ESRF) in Grenoble, France.

**Figure 3:**
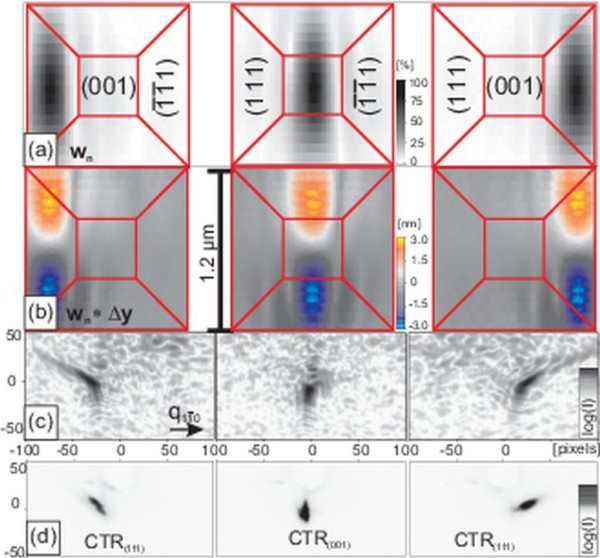
**X-ray nanospot simulation of a scan over a SiGe island.(a)** shows the spot position images, represented by the weighting values *w*_*n*_, whereas in **(b)**, the product of *w*_*n*_with the *y*-component of the internal displacement field is depicted (see text for explanation). **(c)** shows the corresponding RSM simulations near SiGe(004) and the comparison with experimental results **(d)**. The Si substrate reflection has been blocked by a beam stop. Individually excited crystal truncation rods of SiGe(111) side facets can be seen in simulation (**c**) and measurement (**d**).

Near the SiGe(004), reflection RSMs were taken, Figure 3d, using X-ray nanodiffraction at different spot positions on the sample surface by scanning the sample in steps of 600 nm, as illustrated in Figure 3a. The product of the *w*_*n*_ values and displacements in *y* direction, calculated by FEM, is plotted in Figure 3b and illustrates which part of the displacement field actually contributes to the simulation. Both spot position images (Figure 3a,b) are a direct output of the simulation procedure and show a slice from the Si surface.

Using the nanospot simulation method, we calculated RSMs (Figure 3c) for respective positions in real space and at positions in reciprocal space comparable to the measurements (Figure 3d). To perform the simulations, the experimental spot profile of Figure 2 has been used. Although the profile was obtained from a more recent experiment (see section about InGaAs QDMs and [6]), we consider Figure 2 to be closer to reality than an artificial guess. According to the experimental spot size of 200 nm (FWHM), the spot in Figure 2 has been rescaled from 100 up to 200 nm for this purpose. During the experiments on SiGe islands, the ptychography capability was not available yet.

The left and right outer RSMs of Figure 3 show, in simulation and measurement, that individual {111} facet truncation rods have been probed. Since {111} facets have an inclination to the surface of 55°, the facet rods are inclined by 35° from the horizontal axis. Both RSMs in the center of Figure 3c,d exhibit truncation rods originating from the (001) top facet of the SiGe pyramid. The rod appears as a spot only, although broadened in vertical direction, which is due to the coplanar diffraction geometry in connection with a 2D detector where RSMs are inclined against the surface normal. If one compounds all three RSMs of Figure 3c or Figure 3d, one can recognize the butterfly-like diffraction image [2] well known from ensemble averaging experiments on SiGe islands.

It is interesting to mention that a follow-up microdiffraction experiment on 3.2-*μ*m-sized SiGe islands by Diaz et al. showed comparable RSM measurements [21]. In the latter work, the *z*-component of strain has been analyzed for several spot positions.

### SiGe/Si(001) dot molecules

**Figure 4:**
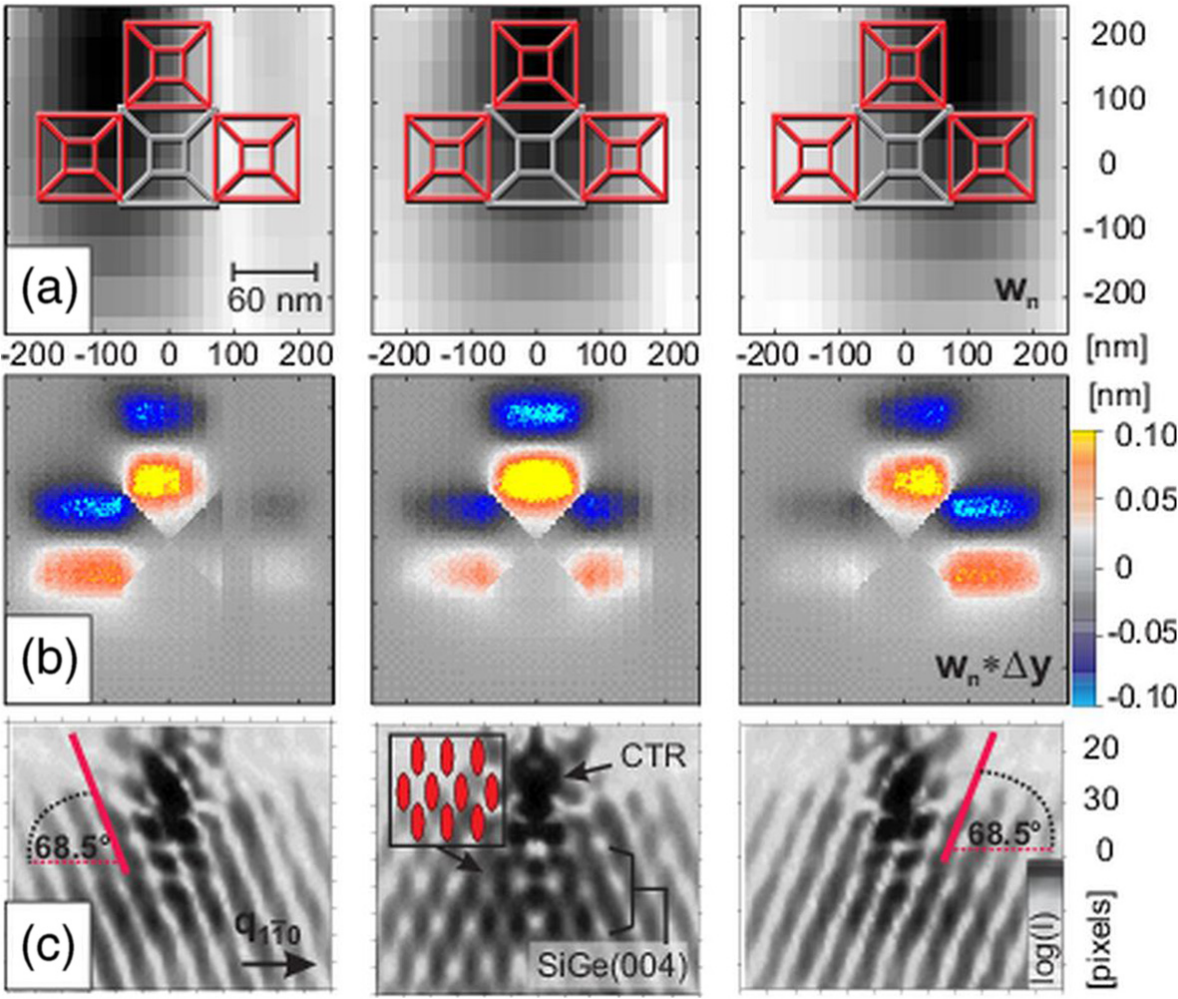
**X-ray nanospot simulation of a scan over a SiGe DMs.(a)** shows the beam spot positions given by the weighting factors *w*_*n*_within the plane of the modelled sample surface. The SiGe dots (red) grown around a pit (grey) are also sketched within (**a**). Weighting factors *w*_*n*_multiplied by the *y*-component of the internal displacement field are shown in **(b)** and reveal the parts of the displacement field that are contributing to the simulation. **(c)** shows the simulated diffraction image near SiGe(004) reflection at the corresponding spot position shown in (**a**).

With SiGe islands, more complex structures can be formed, e. g., small arrangements of several islands self-assembled at pyramid-like pits. As an analogue to chemical molecules consisting of atoms, such arrangements are called dot molecules (DM). We examined Si_0.74_Ge_0.26_/Si(001) DMs consisting of one, two, three, or four dots. A nanodiffraction simulation of a scan over a threefold SiGe DM with a 250-nm X-ray spot is shown in Figure 4. Like in the previous experiment, the images of the weighting factors (Figure 4a) and weighted displacement field (Figure 4b) show the spot positions for the simulation. The corresponding simulated diffraction patterns depicted in (Figure 4c) originate from positional correlation function of the SiGe dots within a DM [4]. From the direction of the lines in Figure 4c (left and right column), it can be derived which part of a DM has been illuminated, and one can distinguish between different kinds of DM consisting of one, two, or four SiGe islands including their azimuthal orientation. Falling lines in the left picture and rising lines in the right picture of Figure 4c give the orientation of the two SiGe islands that are illuminated, and the RSM with honeycomb-like patterns in the center image of Figure 4c is typically obtained from three- or fourfold DMs. However, in case of scanning directly over a fourfold DM also, the left and right RSM in Figure 4c would show honeycomb-like patterns like previously measured at the ESRF [4]. 

**Figure 5:**
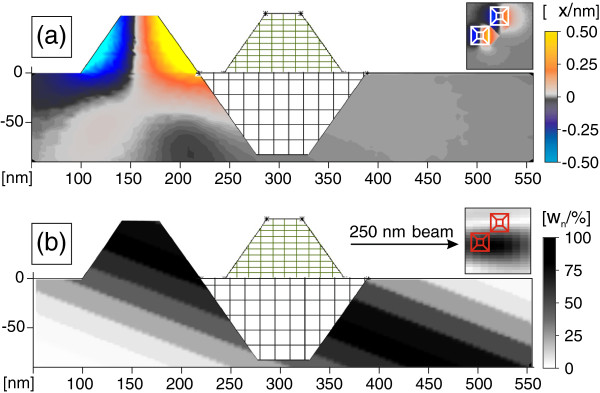
**Internal displacement field and simulated beam path within SiGe DMs.(a)** depicts the *x*-component of the internal displacement field within SiGe DMs and the Si substrate. **(b)** shows the output of the weighting values *w*_*n*_for each scatterer *n* of the material in our model.

Diffraction images induced by a beam spot yield a convolution of the spot shape and the nanostructure shape since, in Fourier transform, both are multiplicands. However, one has to keep in mind that an illuminated 3D volume of the beam path has to be considered. Apart from the 2D spot shape and spot position, this volume depends moreover on the incidence angle and spot size. If parts of larger structures are analyzed, the penetration depth would also play an important role. Due to the strain field below a DM, especially in nHRXRD, the path of the beam through the sample is important, as can be easily understood in Figure 5a,b. The path of the beam could run through specific parts of the nanostructure material and parts of the strain-affected substrate below, or could touch just one of both. For the presented case, the penetration depth of the beam is more than two orders of magnitude larger than the nanostructures and their strain fields within the substrate and, thus, has been neglected. As an example of displacement fields within the substrate, Figure 5a shows a vertical cut through the *x*-component of the 3D displacement field of one DM with two SiGe islands. The beam path is represented by the weighting values *w*_*n*_ as shown for the twofold DM in Figure 5b. Only parts with *w*_*n*_>0 are contributing to the scattering; thus, white areas in Figure 5b are discarded. If the DM configuration of Figure 5 is used as an input for scattering simulations, a RSM with lines comparable to the left picture of Figure 4 is obtained.

For the simulations, the spot profile shown in Figure 2 was used again and rescaled to 250 nm FWHM. The shape of the main maximum of this spot can be considered as round shaped. Side oscillation maxima, see HA and VA in Figure 2a, exhibit amplitude values that hold between 6% and 22% of the main maximum amplitude, and their contribution to the diffraction image is accordantly low. Nevertheless, simulation comparisons between the experimentally gained spot and perfectly round spot shapes showed recognizable differences especially for complex structures like SiGe DMs. This occurs since the FWHM size of the spot was slightly smaller than the illuminated nanostructure, but the strongest side maxima of the spot shape could hit neighboring parts of the material. As a consequence, the spot with side maxima could already lead to a honeycomb-like pattern even if the main spot only hits, e. g., two islands of a threefold DM, whereas the perfectly round spot at the same position leads to line oscillations in the RSM. Comparisons between simulations using a spot without phase variation on the one hand and with phase variation on the other hand revealed only minor distinctions that were, in our case, negligible.

**Figure 6:**
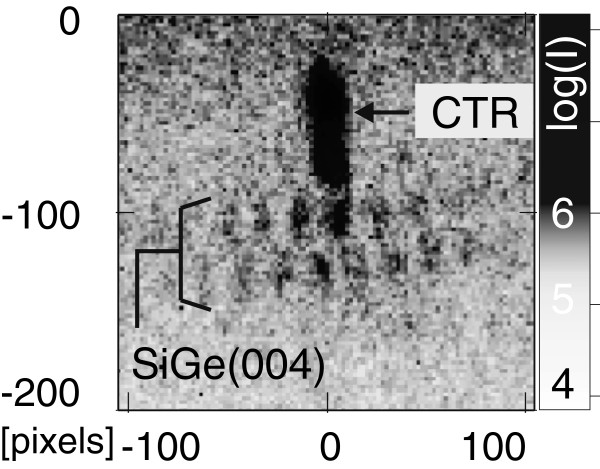
**SiGe(004) reflection acquired in a nanodiffraction experiment on SiGe/Si(001) DMs.** The 2D detector frame shows the crystal truncation rod (CTR) of the (001) surface and the diffuse intensity with positional correlation peaks around the SiGe(004) reflection (SiGe).

Since the signal of the Si substrate reflection is much more intense than the reflection from the small amount of SiGe material, it is much easier to find and measure the substrate reflection instead of the SiGe reflection. Consequently, in most nHRXRD measurements at comparable structures, only the substrate reflection could be measured. However, in terms of the positional correlation within dots, both reflections display identical patterns if the beam directly hits the entire DM. This can be ascribed to the fact that the strain field distribution in the substrate underneath a DM carries similar information about the dot positions. To be precise, further simulations (not shown) reveal that Si(004) and SiGe(004) reflections can indeed exhibit different diffraction patterns within one RSM: This occurs if the beam hits, e. g., two SiGe islands of a threefold DM and coincidentally illuminates the underlying strain field of all three islands. In this case, around the Si(004) reflection, a honeycomb-like pattern of three islands becomes visible, but at the SiGe(004) reflection, a pattern with diagonal lines appears since the third SiGe island itself was not illuminated directly. In a nanodiffraction experiment, we measured the diffusely scattered intensities at one individual SiGe DM that also reveal similar patterns of substrate and structure reflection: In Figure 6, a RSM near the SiGe(004) reflection is shown where a honeycomb-like pattern becomes visible, which is reminiscent of the pattern at the Si(004) substrate reflection investigated in reference [4]. The measurements at the Si(004) reflection have been actually performed at the same SiGe DM like the RSM presented in Figure 6 (beamline ID13/ESRF, 250-nm X-ray spot focused by Kirkpatrick-Baez mirrors at 12.4 keV). In Figure 6, the diffuse SiGe reflection appears laterally elongated due to the {111} facet rods of the islands comparable to that of Figure 3b,c. 

### InGaAs/GaAs(001) quantum dot molecules

**Figure 7:**
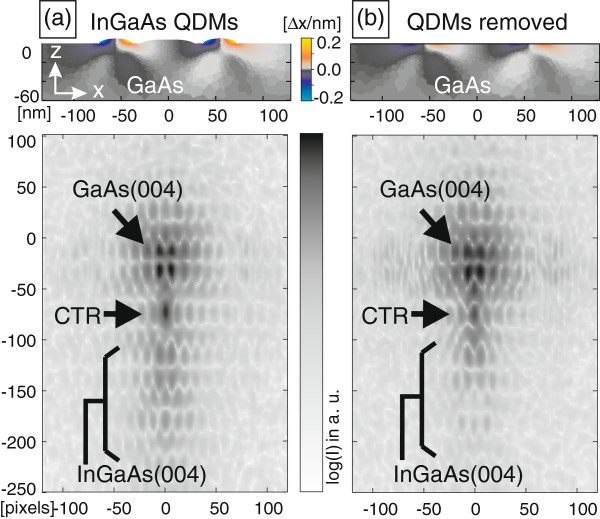
**Displacement field within InGaAs QDMs and related X-ray nanodiffraction simulation.(a)** shows the vertical slice through the *x*-component of the displacement field within In_0.3_Ga_0.7_As/GaAs QDMs calculated by FEM and the corresponding scattering simulation near the GaAs(004) and InGaAs(004) reflections. **(b)** shows a comparable slice like that of (a) but after removing all 3D data of the InGaAs QDMs directly before running the scattering simulations shown below. The FEM strain field calculations have been performed before cropping the model data.

Finally, we applied the simulation method to a very shallow In_0.3_Ga_0.7_As/GaAs(001) QDMs. A diffraction on these structures using a 100-nm X-ray beam spot also revealed the positional correlation of the QDs within the molecules [6]. In this experiment, the GaAs(004) substrate reflection has been measured for intensity reasons. The examined QDMs consist of six In_0.3_Ga_0.7_As QDs self-assembled around a GaAs dot on the GaAs surface. Figure 7a shows a slice through the 3D displacement field within the GaAs substrate and two of the six In_0.3_Ga_0.7_As QDs (the *x*-component of the displacement is displayed). The highest displacement can be found within the QDs, but also at the interface towards the substrate, a quite complex displacement field in the deeper regions of the substrate is visible. The corresponding GaAs(004) nanospot scattering simulations at this model are depicted below the picture Figure 7a of the displacement field. For a closer description of the sample system and related diffraction measurements, see also reference [6]. 

To examine the influence of the strained substrate on the diffraction image, we ran additional scattering simulations within the substrate material only. After performing FEM calculations to get the internal elastic strain field, we cut off the actual In_0.3_Ga_0.7_As QDs from the model data and generated a RSM by diffraction simulation (Figure 7b). Due to less material with high lattice parameter, the intensity at lower diffraction angles (smaller reciprocal coordinates) becomes much weaker. Only the strained part of the substrate leads to a broadening of the substrate reflection towards the InGaAs(004) reflection. However, it is evident that the patterns of the whole visible intensity distribution are still identical with that of Figure 7a. This comparison clearly indicates that the positional correlation of the QDs can be measured indirectly by measuring the strained substrate instead of the QDs themselves. The patterns then originate from the correlation function not of the QDs but of the change in the strain field induced by the nanostructure directly below the dots. Although this result holds also for a similar cutting procedure on a model with SiGe DMs, the simulation on a model of InGaAs QDMs provides a more direct interpretation without any substrate shape anomalies such as pits. For the simulations, the measured illumination function, Figure 2, obtained from the corresponding experiment [6] was taken to calculate the weighting values *w*_*n*_.

## Conclusions

We developed a way to extend kinematical scattering calculations for nanofocus applications by including an X-ray spot simulation and considering the geometric situation in experiments with area detectors, which are preferably applied in modern X-ray diffraction measurements. The simulation procedure has been applied to different sample systems, and the simulated RSMs were compared with experimental results of scanning X-ray nanodiffraction. We observed that positional correlation between quantum dots within dot molecules can be examined directly or indirectly by acquiring diffuse intensities near Bragg reflections of the structure material or of the substrate material instead. For nHRXRD, the actual path of the beam through the analyzed material is proven to be essential for the diffraction patterns at the recorded reflections. Thus, the presented simulation method may help to distinguish between shape-, ordering-, and strain-induced effects within RSM of nanodiffraction experiments on semiconductor nanostructures.

## Abbreviations

CTR: crystal truncation rod; DM: dot molecule; FEM: finite element method; FWHM: full width half maximum; nHRXRD: nanofocus high resolution x-ray diffraction; QD: quantum dot; QDM: quantum dot molecule; RGB: red green blue (color values); RSM: reciprocal space map; 2D: two-dimensional.

## Competing interests

The authors declare that they have no competing interests.

## Author’s contributions

MD participated in the X-ray nanodiffraction experiments, performed the scattering simulations, and drafted the manuscript. MH conceived of and participated in the experimental studies on SiGe islands. JP and RH participated in the experiments and aligned the X-ray optics. CS conceived of the X-ray optics and coordinated its installation and alignment. SS and MB participated in the experiments. All authors read and approved the final manuscript.
